# Computational modelling of social cognition and behaviour—a reinforcement learning primer

**DOI:** 10.1093/scan/nsaa040

**Published:** 2020-03-30

**Authors:** Patricia L Lockwood, Miriam C Klein-Flügge

**Affiliations:** Department of Experimental Psychology, University of Oxford, Oxford OX1 3PH, United Kingdom; Department of Experimental Psychology, Wellcome Centre for Integrative Neuroimaging, University of Oxford, Oxford OX1 3PH, United Kingdom; Department of Experimental Psychology, University of Oxford, Oxford OX1 3PH, United Kingdom; Department of Experimental Psychology, Wellcome Centre for Integrative Neuroimaging, University of Oxford, Oxford OX1 3PH, United Kingdom

**Keywords:** computational modelling, reinforcement learning, social, reward, model fitting, model selection

## Abstract

Social neuroscience aims to describe the neural systems that underpin social cognition and behaviour. Over the past decade, researchers have begun to combine computational models with neuroimaging to link social computations to the brain. Inspired by approaches from reinforcement learning theory, which describes how decisions are driven by the unexpectedness of outcomes, accounts of the neural basis of prosocial learning, observational learning, mentalizing and impression formation have been developed. Here we provide an introduction for researchers who wish to use these models in their studies. We consider both theoretical and practical issues related to their implementation, with a focus on specific examples from the field.

## Introduction

Learning about actions and outcomes fundamentally shapes social cognition and behaviour. For example, to help others, we need to know how our decisions reward or avoid harming someone else. Before we decide what to choose for ourselves, we can engage in observational learning by watching the good or bad things that happen to other people, and we can infer others’ mental states by tracking their actions and outcomes over time. But how do we form associations between actions and outcomes when they occur in a social context? And are the brain areas involved in social learning uniquely ‘social’ or do they reflect domain-general processing shared with other cognitive faculties? One of the most important influences on psychology, neuroscience and economics has come from associative or reinforcement learning theory that precisely and mathematically describes how decisions are paired with outcomes over time (Sutton and Barto, 1998; Dayan and Balleine, 2002).

Inspired by early behaviourist work on classical conditioning (Pavlov, 1927; Sutton and Barto, 1998), Rescorla and Wagner (1972) proposed their learning model which described how learning occurs via a prediction error, the discrepancy between what we expect to happen and what actually happens. This error correction learning process can be described mathematically. The idea is that the expectations of future reward (or avoidance of punishment) (V_t + 1_) should be a function of current expectations (V_t_) and their discrepancy from the actual outcome that is experienced (r_t_), known as the prediction error (PE_t_), multiplied by a learning rate (a). The prediction error is simply the size of the difference in the outcome we actually receive (r_t_) and the expectation of that outcome (V_t_). The prediction errors’ scaling by a subject-specific learning rate modulates the influence of the prediction error on learning:

$$ {\mathrm{V}}_{\mathrm{t}+1}={\mathrm{V}}_{\mathrm{t}}+\mathrm{a}\,{}^{\ast} {\mathrm{PE}}_{\mathrm{t}} $$

where

$$ \mathrm{PE}={\mathrm{r}}_{\mathrm{t}}-{\mathrm{V}}_{\mathrm{t}} $$

**In simple language:**

*Expectations on the next trial* = *the expectation on the current trial* + learning rate^*^prediction error (reward – *current expectation)*

Perhaps central to questions of social reinforcement learning, these prediction errors can be social in nature, i.e. ‘social’ prediction errors, such as the expectation that my action will help someone *vs* the outcome that it did or did not (Lockwood *et al.*, 2016), or my expectation that I will be liked by someone else and the outcome that I was or was not (Will *et al.*, 2017; Yoon *et al.*, 2018). Whilst brain areas classically associated with reinforcement learning such as ventromedial prefrontal cortex and ventral striatum have been linked to processing both social and non-social prediction errors (discussed in more detail below), there may also be social prediction error signals in regions somewhat specialized for social processing (i.e. that do not typically respond to reward processing in general). These regions include the anterior cingulate gyrus (Behrens *et al.*, 2008; Apps *et al.*, 2015; Lockwood *et al.*, 2018), subgenual anterior cingulate cortex (Lockwood *et al.*, 2016; Will *et al.*, 2017), temporo–parietal junction and dorsomedial prefrontal cortex (e.g. Hampton *et al.*, 2008; Koster-Hale and Saxe, 2013; Hill *et al.*, 2017). A more extensive discussion about the distinction between brain regions coding social and non-social prediction errors is covered in several other recent reviews (e.g. Joiner *et al.*, 2017; Wittmann *et al.*, 2018; Olsson *et al.*, 2020; Suzuki and O’Doherty, 2020).

**Fig. 1:**
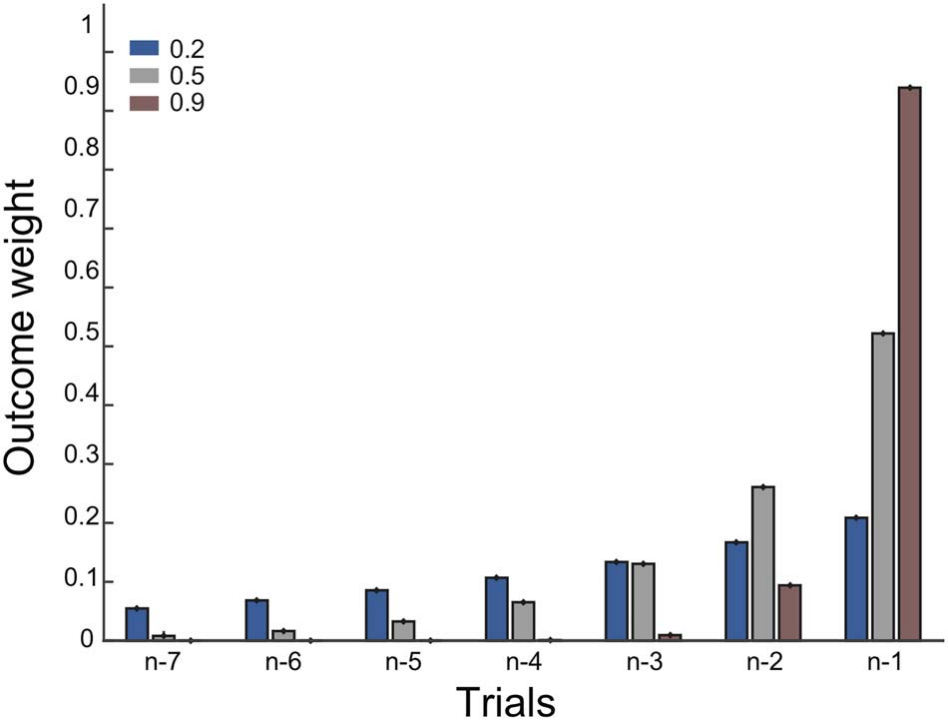
The influence of recent outcomes onto choice, for different learning rates. Shown is the influence of the outcomes received on the last seven trials for making a choice on the current trial, for three different learning rates. Red shows a hypothetical participant with a learning rate of 0.9. This learner updates strongly based on recent outcomes. There is a strong influence of the outcome on the previous trial (*n* − 1), and a weaker influence of the outcome received two trials back (*n* − 2) but virtually no influence of earlier outcomes. A learner with smaller learning rates, here shown for 0.5 (grey) or 0.2 (blue), shows increasingly longer-lasting influences of outcomes received on trials further back from the current trial.

In the context of reinforcement learning, as shown in the update equation above, the prediction error is scaled by the learning rate for updating expectations. The learning rate can also differ according to the context in which learning occurs (discussed in further detail in section ‘one parameter or many parameters’ below). An example of how past outcomes influence a current choice is illustrated for three different learning rates in Figure 1.

Importantly, the utility of reinforcement learning (RL) models has been bolstered by their neural plausibility—the discovery that phasic activity of dopamine neurons in the midbrain encode a prediction error (Schultz, 2007, 2013). Not only did this model-derived updating signal have a distinct neural correlate, but it arguably has transformed classical neuroimaging analysis techniques (Behrens *et al.*, 2009). Whilst classical fMRI studies had to rely on a subtraction-based design where average activity for two categories was contrasted (e.g. faces *vs* houses), now there was a method that produced parametric values on every single trial that could be used to look for areas of the brain that covary with predictions from the model over time. In other words, this advance in experimental design for the first time provided a handle on the precise computation occurring in a brain area. Moreover, model-based fMRI could potentially help bridge different levels of explanation from the cognitive and behavioural to the neural.

Studies in the field of social neuroscience have begun to apply these models to understand how and whether quantities predicted by RL are represented in the brain during social situations (Behrens *et al.*, 2008; Hampton *et al.*, 2008; Burke *et al.*, 2010; Suzuki *et al.*, 2012; Seo *et al.*, 2014; Apps *et al.*, 2015; Hackel *et al.*, 2015; Sul *et al.*, 2015; Kumaran *et al.*, 2016; Lockwood *et al.*, 2016, 2018, 2019; Spiers *et al.*, 2016; Wittmann *et al.*, 2016, 2018; Zaki *et al.*, 2016; Cheong *et al.*, 2017; Hill *et al.*, 2017; Will *et al.*, 2017; Charpentier and O’Doherty, 2018; Konovalov *et al.*, 2018; Lindström *et al.*, 2018; Lockwood and Wittmann, 2018; Yoon *et al.*, 2018; Farmer *et al.*, 2019; Zhang *et al.*, 2019; Olsson *et al.*, 2020). The implementation of these models has already provided important new insights into multiple aspects of social behaviour. For example, many studies have documented how medial prefrontal cortex often responds to contrasts of Self > Other, in terms of referential judgement and even processing of faces, leading some authors to suggest that mPFC is critically involved in self-representation (Kelley *et al.*, 2002; Northoff *et al.*, 2006; Sui and Humphreys, 2015). However, we recently showed that using a parametric approach this same portion of ventral mPFC in fact tracks associative learning relevant to ourselves, friends and strangers, on every trial, significantly above chance. We were able to replicate an overall subtraction effect of Self > Stranger but could additionally show that this area in fact held representations of all three agents in parallel. This finding would not be possible in a subtraction design where the individual parameter estimates themselves are not interpretable (Lockwood *et al.*, 2018).

**Fig. 2:**
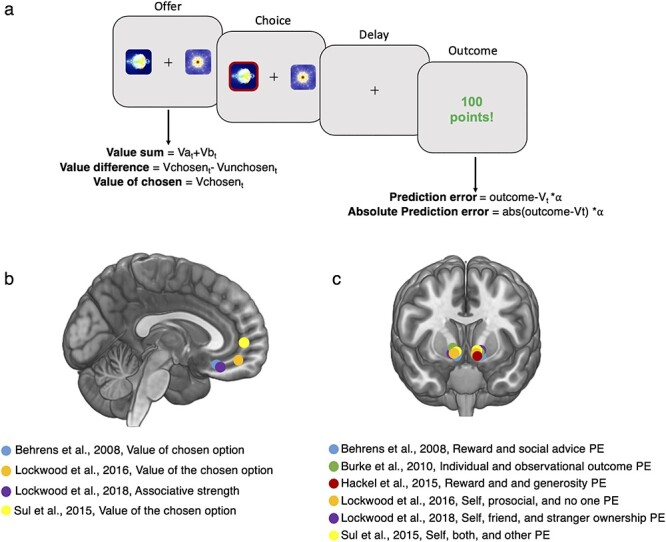
Schematic of task structure from a two-armed bandit task and associated neural signals from social reinforcement learning studies. (A) Example of a two-armed bandit task. At the offer stage, two options are presented that are probabilistically associated with a reward. In some experiments, they could also be associated with different magnitudes of reward, or both reward probability and magnitude could be varied. Participants learn by trial and error which of the two options provides a better outcome. At the time of the offer, various quantities can be modelled, including the associative strength between the picture and the outcome, the value sum, value difference or value of the chosen option. At the time of the outcome, either the signed prediction error which codes the expectedness of the outcome or an ‘absolute’ prediction error could be modelled. The absolute prediction error ignores the sign (positive or negative) of the prediction error but quantifies the overall unexpectedness of the outcome. (B) Studies of social reinforcement learning that have reported tracking of value/associative strength signals in ventromedial prefrontal cortex at the time of choice overlaid on an anatomical scan of the medial surface. (C) Studies of social reinforcement learning that have reported tracking of prediction errors that overlap in social and non-social situations at the time of an outcome in the ventral striatum overlaid on an anatomical scan. Note that a meta-analysis from NeuroSynth shows that the most robust response to the term ‘prediction error’ is in the ventral striatum (overlap from 93 studies). PE, prediction error.

Another example from parametric reinforcement learning fMRI studies is that responses to prediction errors in ventral striatum appear to be non-specific, that is, responses in this area track PEs in both social and non-social contexts when directly compared (Behrens *et al.*, 2008; Burke *et al.*, 2010; Sul *et al.*, 2015; Lockwood *et al.*, 2016, 2018) (Figure 2C). Such a pattern is consistent with ventral striatum encoding a domain-general learning mechanism or domain-general reinforcement (Schultz, 2007, 2013; Daw *et al.*, 2011; Klein-Flügge *et al.*, 2011), rather than supporting the idea that this region encodes how rewarding it is, or warm glow associated with helping another person. These are just a few of the examples of how a parametric analysis approach might lead to new insights into human social behaviour.

In the next sections, we discuss the application of different types of reinforcement learning models in social neuroscience studies as well as practical methodological considerations for researchers wishing to apply these models to their own data. We focus on neuroimaging studies that have used RL models in this article. Parameters from RL models can also be applied to data acquired using other types of methods (EEG, MEG, behavioural parameters in lesion studies, pharmacology and TMS), and therefore this guidance could also apply to those modalities. Similarly, these guidelines should be relevant to any researcher wishing to apply reinforcement learning to their studies even in non-social domains, including studies in healthy people and those with neurological and psychiatric disorders (Friston *et al.*, 2014; Lockwood, 2016; Scholl and Klein-Flügge, 2018). However, they may also wish to consult many other reviews and excellent guidelines on the topic (Daw and Doya, 2006; Dayan and Niv, 2008; Samson *et al.*, 2010; Daw, 2011; Zhang *et al.*, 2019).

## Applying reinforcement learning models in studies of social neuroscience: theoretical considerations in reinforcement learning

### What type of reinforcement learning model should I chose?

A first question when designing a study to investigate a social neuroscience question with RL models is how best to design the experiment and what type of RL model to use. Here we briefly review some of the most common RL models used in the field. For advice on general experimental design, we refer to another review that covers this topic (Wilson and Collins, 2019). The simplest reinforcement learning model allows for two parametric values to be calculated trial-by-trial that can be correlated with neural responses. The first of these are the quantities associated with expectations, often termed associative strength, value or expected value (‘V_t_’ in the equation in the previous section). The second of these is known as the prediction error (Figure 2A–C) (‘PE’ in the equation in the previous section).

A clear illustration of the difference between these two quantities can be seen through an example of a two-armed bandit task (Figure 2A). In this task, two options are presented, A and B. A is associated with a high probability of reward and B is associated with a low probability. By trial and error the participant learns which of the two options is most likely to deliver a reward. The expectations are calculated at the time of the choice between A and B. There are now several options for creating parametric regressors based on these expected values of A and B. The researcher can decide whether the most relevant way to model this quantity is as the value difference between the two options (A and B) on every trial, the value of the chosen option, the value of the chosen option minus the value of the unchosen option or the sum/mean of the values on offer (Hunt *et al.*, 2012).

It is also important to test for areas that inversely code value difference (parametric value at the second level of an fMRI analyses of −1) as several studies have reported areas that negatively track value, that is, they increase their response when the value difference is small and suppress their response when the value difference is large (Scholl *et al.*, 2015; Klein-Flügge *et al.*, 2016; Chong *et al.*, 2017; Lockwood *et al.*, 2018; Piva *et al.*, 2019). Neurally, previous studies have suggested that these quantities are often associated with a signal in the ventromedial prefrontal cortex in both social (Nicolle *et al.*, 2012; Zhu *et al.*, 2012; Boorman *et al.*, 2013; Sul *et al.*, 2015; Lockwood *et al.*, 2016, 2018; Apps and Ramnani, 2017; Lockwood and Wittmann, 2018; Fukuda *et al.*, 2019; Piva *et al.*, 2019) and non-social studies (Kable and Glimcher, 2007; Hunt *et al.*, 2012; Levy and Glimcher, 2012; Bartra *et al.*, 2013). Whether the sign of value tracking is functionally meaningful is highly debated (e.g. positive *vs* negative tracking of value) with differences in sign perhaps reflecting whether the signal is tracking value, difficulty, salience or arousal (Bartra *et al.*, 2013). In studies that do not involve learning and where all information about choice options is displayed on the screen, the value difference can be computed without the need for a learning/RL model and usually involves similar neural signals (Nicolle *et al.*, 2012; Klein-Flügge *et al.*, 2016; Apps and Ramnani, 2017; Piva *et al.*, 2019). Note that in such tasks, behavioural analysis will in many cases still involve model fitting of other types of models, for example, economic choice models of risk and delay (Ruff and Fehr, 2014).

The second signal that is often calculated is the prediction error, the difference between the outcome and the expectation (Schultz, 2007). Considerations when studying a prediction error signal include interpreting the sign of the prediction error. Often brain areas will be found positively correlating with the PE signal (areas that increase their response when the outcome is positive and decrease their response when the outcome is negative). However, as with the value difference or value coding, it is also important to test for areas that show the reverse pattern, that is, they increase their signal when the outcome is negative/neutral and decrease their signal when the outcome is positive. Another consideration is whether to examine ‘absolute’ prediction errors that code for the general unexpectedness of an outcome regardless of being positive or negative or test for a ‘signed’ prediction error. Finally, with the simple RL model, it can also be informative to appropriately characterize the learning rate, which is usually a single subject-specific parameter. The learning rate can then be correlated with the parametric values from the model to assess if individual differences in learning correlate with parametric values of neural activity. Neurally, prediction error signals at the time of outcome are most commonly associated with tracking in the ventral striatum in both social (Behrens *et al.*, 2008; Burke *et al.*, 2010; Boorman *et al.*, 2013; van den Bos *et al.*, 2013; Sul *et al.*, 2015; Lockwood *et al.*, 2016, 2018; Hertz *et al.*, 2017; Wittmann *et al.*, 2018) and non-social studies (O’Doherty, 2004; Daw *et al.*, 2011; Klein-Flügge *et al.*, 2011; O’Doherty *et al.*, 2017). In support of a key role of ventral striatum in tracking a general prediction error signal, we conducted a meta-analysis in NeuroSynth using the term ‘prediction error’ which showed that across 93 studies, ventral striatum was the most robustly activated brain area.

Aside from this simple RL model, there are several derivations of the model that may be particularly relevant in studies of social neuroscience. Observational learning—learning from the actions and outcomes of others—has been characterized within a reinforcement learning framework through ‘observational action prediction errors’ and ‘observational outcome prediction errors’ (Burke *et al.*, 2010). Social computations might also be described in a case where a person should estimate an expectation of another person’s action in order to update their own action in a strategic interaction. For example, in the inspection game a worker needs to decide to work or not work on the basis of their expectation that an employer will inspect or not inspect (Hampton *et al.*, 2008; Yoshida *et al.*, 2010; Hill *et al.*, 2017). These social signals have been linked to activity in the temporo–parietal junction and dorsomedial prefrontal cortex, areas typically associated with theory of mind-related computations in classical studies (Saxe and Kanwisher, 2003; Younga and Saxe, 2008). Finally, models can be chosen of prosocial learning where the action is always from the participant himself or herself, but the outcome is varied to different social agents. This can create a ‘prosocial prediction error’ where participants learn which of their actions results in reward or avoidance of punishment for others (Lockwood *et al.*, 2016, 2019). In this case more complex RL models can be used, such as those that distinguish between ‘model-free’ and ‘model-based’ learning. Model-free learning is the term used to describe simple RL learning where actions and outcomes are paired based on reinforcement. In contrast model-based learning takes into account the structure of the environment and specifically how actions and outcomes are mapped. We recently showed that people were more ‘model-free’ when learning about avoiding harm to others, and this was reflected in multiple neural signals of model-free learning (Lockwood *et al.*, 2019).

### Practical methodological considerations in reinforcement learning: model fitting and parameter estimation

Model fitting can at first appear a bit like a ‘black box’: when feeding in choices of an individual participant, the optimization algorithm spits out what is hoped to be the best parameter estimate. In the next few sections, we will try and unpack what exactly is happening within this ‘black box’. We will also outline how, instead of blindly believing model fitting results, some basic checks can help ensure robustness and validate the model fitting procedure.

#### What is a parameter?

**Fig. 3:**
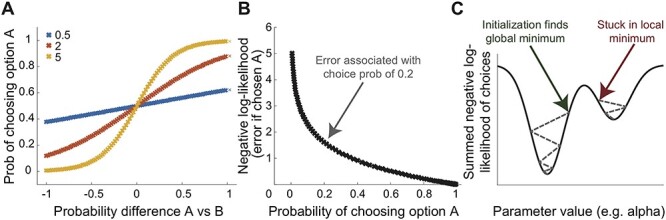
Softmax temperature, negative log-likelihood and error minimization: (A) obtained choice probabilities are shown for three different values of the inverse temperature parameter of the softmax function (‘beta’). Larger inverse temperature values correspond to a steeper function and thus less noisy choices. Note that the range of the beta values will depend on the range of the ‘decision variable’, here the probability difference between A and B which can vary between [−1, 1]. It can be helpful to scale decision variables in comparable ranges so that the scale of the temperature parameter becomes interpretable (note that only multiplicative scaling, but no additive shifting, should be applied to decision variables). (B) The choice probabilities are log-transformed and inverted (−log(choiceProb)) to obtain the negative log-likelihood of each choice. This not only makes it practically possible to compute the likelihood (product) of all choices because the log of the product is the sum of the log-transformed values. But it also means that very wrong predictions (e.g. a low 0.2 predicted probability of choosing option A when the participant actually choses option A) will be given a stronger weight in the overall error. (C) The summed negative log-likelihood of all choices needs to be minimized to obtain the best fit. This is done internally by fitting algorithms by varying the parameter values (here just alpha) until the parameter value that is associated with the minimum error is found. Because of local minima, it is sometimes important to run fitting algorithms with multiple parameter starting values.

In contrast to variables like the prediction error, which are estimated for every trial of an experiment, the parameters fitted in a computational model are categorically different in that they are represented by only a single number per experimental session. For example, usually experiments assume only a single learning rate (α) per session, and the value for that number ranges between 0 and 1. The learning rate was part of the equation described at the beginning of this article. In addition, most learning experiments fit an additional parameter that captures, across the entire experiment, the noisiness or stochasticity of an individual’s choices. This parameter is referred to as the inverse temperature (‘beta’, (β)) and controls the steepness of the softmax function. This function translates the value difference between two options A and B into the probability of choosing option A (a quantity needed for model fitting as described below). It is shown for three different values of beta in Figure 3A. Note that beta will scale with the range of the values on the x axis (i.e. the value difference).

#### What is parameter fitting?

In general, fitting algorithms try and minimize the error between the prediction achieved with a particular combination of parameters (e.g. learning rate alpha (α) and inverse temperature softmax beta (β)) and the true data. For choice data, because the decision variable is fed through the softmax function (Figure 3A), as explained above, each trial is associated with a choice probability or in other words a likelihood that this choice would have been made given the combination of model parameters. The question that follows is: what is the likelihood of all choices together given this parameter combination? The likelihood of multiple events is calculated using the product of each of the individual observation’s likelihood. For example, the probability for tossing heads three times in a row is 0.5 ^*^ 0.5 ^*^ 0.5 = 0.125. But multiplying many small numbers (e.g. here the choice probabilities associated with something like 200 trials) quickly becomes computationally imprecise because the resulting product becomes very small. A simple trick is therefore used: to calculate the error, the logarithm of the product of all choice probabilities is computed which is the same as the sum of the log-transformed probabilities (log(a ^*^ b) = log(a) + log(b)). Using the log-transformed choice probabilities has another desirable effect, namely, that completely opposite (and thus wrong) predictions more heavily influence the error term than predictions close to the true choice (Figure 3B). Finally, because the aim is to find the parameter combination that maximizes the likelihood of the data, but most algorithms are built to ‘minimize’, the error term that is returned is the *negative log-likelihood*.

Establishing the parameter combination associated with the maximum likelihood of a set of choices can be achieved using many different toolboxes and programming languages. It is not our aim here to cover the precise algorithms that achieve this minimization (e.g. Nelder–Mead simplex algorithm used by fminsearch in Matlab). From a more practical standpoint, however, it is worth checking whether the maximum likelihood (and associated set of parameters) is the same when initializing the model fitting from different parameter starting values. If it is not, it means that the algorithm might have gotten stuck in local minima, rather than finding the global minima in all cases (Figure 3C). This can happen particularly in complex models with many parameters because the parameter space becomes multidimensional or in situations with few trials. In such cases, multiple initializations from a grid of starting values can be used, and the parameters associated with the initialization that leads to the maximum likelihood (minimum negative log-likelihood) are reported. Independently, a grid search, which evaluates the function at a grid of parameter values (without minimization) and saves the negative log-likelihood for each combination, can be helpful for developing an intuition for the landscape, but it is computationally expensive. Below we give more advice on how to check whether the parameters can be estimated reliably.

**Fig. 4:**
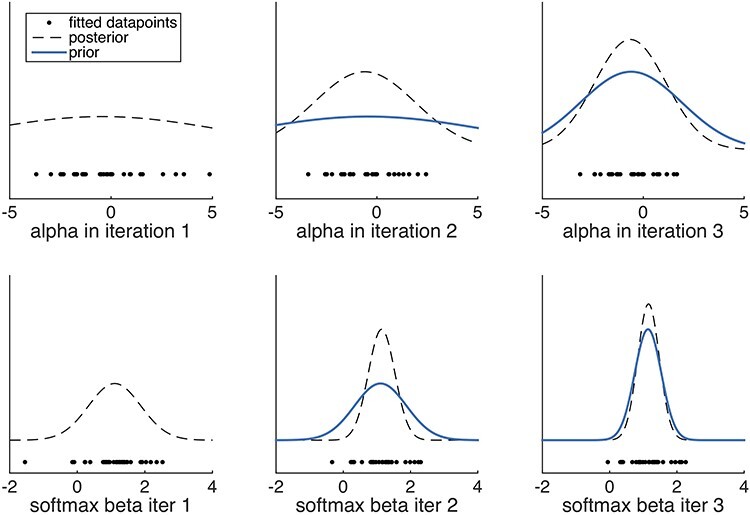
Illustration of the effects of hierarchical fitting for two parameters alpha (learning rate) and beta (softmax temperature). Left column: shown are the initial alphas and softmax betas obtained using a flat prior. The posterior obtained from the first iteration is shown in the dashed black line. Middle column: in this iteration, the posterior from iteration 1 becomes the prior (blue). This ‘pulls in’ several estimates that were previously at the more extreme ends of the range for alpha and beta. The posterior from this second iteration is sharper. Right column: Using the posterior from iteration 2 as the prior in iteration 3, there are only smaller changes in parameter estimates in this third iteration. More iterations would follow (not shown) until the algorithm converges. Note that both parameters are shown in a range between [-Inf, Inf] here. They are transformed to values between [0, 1] for alpha or positive values for beta using the transformations 1/(1 + exp(−alpha)) and exp(beta), respectively.

Despite all the above efforts, fitting of individual participant’s data can still be noisy and variable and involve outliers. There are many reasons for this, for example, restricted time windows during fMRI studies only allow for limited numbers of trials, strategies differ between participants, some participants produce noisy data, etc. *Hierarchical fitting* offers a solution to this; the aim here is to maximize the likelihood of the choice data whilst ensuring everyone’s fitted parameters are drawn from a common Gaussian distribution (per parameter). In other words, the goal is not merely to find the parameters that give the maximum likelihood of the data, but what is maximized is the product of the likelihood of the data given the parameters *and* the likelihood of the parameters given the distribution of parameters (e.g. Huys *et al.*, 2011; Huys *et al.*, 2012). The prior distribution over the parameters, over multiple iterations, moves outlier fits closer to the mean and thus serves to regularize the resulting parameters (Figure 4). For a more detailed and mathematically precise explanation, see: (Daw, 2011; Huys *et al.*, 2011, 2012) or the STAN documentation (Sorensen and Vasishth, 2016; Carpenter *et al.*, 2017). For examples of social reinforcement learning studies using this approach see: (Lockwood *et al.*, 2016, 2019; Diaconescu *et al.*, 2017; Hill *et al.*, 2017).

Ultimately, one main advantage of using computational models to study social cognition is that once model parameters have been fitted, they can be used to generate trial-by-trial estimates for each individual, for example, for prediction errors or the subjective values of choice options. These can then be related to behavioural (e.g. reaction time) or neural data (e.g. BOLD fMRI). In case of large parameter ranges or outliers in the fitted parameters, it is worth considering using the mean fitted parameters from all participants to generate trial-by-trial predictors. Sometimes this has been found to be more robust (Daw *et al.*, 2006, 2011; Schonberg *et al.*, 2007; Lockwood *et al.*, 2018, 2019). This can also be worth considering when some participants have very small learning rates close to 0, meaning their parametric regressors are almost flat and cannot be used to meaningfully explain variations in BOLD activation (this is less likely to be problematic when doing a hierarchical fit; but even then, the group-level parameter can be used). In general, we recommend to visually inspect and normalize (z-transform) parametric predictors before inclusion in a behavioural or brain general linear model (GLM), unless normalization is already implemented as part of the software package. It is also worth noting that small changes in parameter values (e.g. using the group mean rather than the individual’s set of parameters) often produce highly correlated trial-by-trial regressors and consequently similar results. Again, correlations can be inspected before deciding whether to use individual or group parameter estimates.

#### One parameter or many parameters?

Central to questions of computational modelling is the number of parameters required to appropriately explain behavioural data. This consideration may particularly affect social neuroscience studies where researchers want to capture some aspect of social compared to non-social learning or interactions between different parameters (such as learning rates) with the same computational structure but possibly different values, such as learning rates for self being higher than learning rates about other people. This is also a consideration for non-social studies where there is an increasing appreciation that different learning rates may be necessary to explain learning from positive *vs* negative/neutral outcomes (e.g. Costa *et al.*, 2016; Eldar *et al.*, 2016; Lockwood *et al.*, 2019). Determining the utility of including an additional parameter, e.g. to explain different learning patterns, can be done using simulated data and model comparison. This as well as another simple check to ensure the fitted parameters can be trusted is discussed in the next section.

### Validating the model fitting procedure: simulating data and regression analyses

Before starting a new study, one should consider which model, or variants of a model, is likely to describe behaviour in the task and would be suitable to answer the scientific question of interest. In many cases, there are already models available that can be used or adapted (some of which we described above). Once a putative model has been established, it is recommended that simulated (also referred to as synthetic) data is generated. The advantage of doing this is that the ground truth is known in simulated data. For example, choices are produced for a virtual agent with a learning rate alpha = 0.2 and an inverse temperature beta = 3. What this means is that the experimenter knows and has full control over exactly which parameters are used to generate the data. This, of course, is never true for data collected from participants.

Once simulated, synthetic data can be treated like data collected from participants and the same model fitting procedures can be applied. The crucial difference is that because we know in advance which result we expect, we can check how close the fitted values are to the true values. In the above example, fitting the choices of the virtual simulated agent should result in parameter estimates close to 0.2 for alpha and close to 3 for beta. In a learning paradigm, synthetic choice data would be generated using a range of different alphas, e.g. ranging from 0.1 to 1, and a learning rate would be fitted to all these virtual agents to ensure that the correct learning rate parameters can be recovered. This is repeated for all parameters of interest. *Parameter recovery* thus refers to the relationship, usually measured in terms of Pearson’s correlation coefficient, between true (simulated) and recovered parameters (e.g. Lockwood *et al.*, 2019). The recovered parameters are those obtained from fitting the model to the simulated data. Whilst there is no hard boundary in terms of what constitutes ‘sufficient’ and ‘insufficient’ recovery, the stronger the correlations, the more convincing it is that parameters can be estimated robustly. Parameter recovery can be repeated for several putative models. If it fails, this could have multiple reasons. Often, it means that there is insufficient data to estimate the number of parameters or that the parameters are not sufficiently independent. Alternatively, there could be insufficient variation in the critical task manipulations on which the parameters load (e.g. fluctuations in the probability to estimate an adaptive learning rate). Thus, parameter recovery can be taken as an indication that a task schedule or its duration is sufficient. Finally, simulations can be used to test that the presence or absence of an effect would be recovered correctly during model fitting. For example, can a different learning rate when learning for oneself compared to another agent be recovered from the simulated data that does have this effect inbuilt, but *not* be recovered when both agents were given the same learning rate during the simulation? (‘model falsification’; Palminteri *et al.*, 2017; see also Melinscak and Bach (2019) for details on task optimization in associative learning studies) Note that parameter recovery is particularly important when designing a new experimental paradigm or new trial schedules, as compared to using previous tasks that might already be validated. The importance of this step is a relatively recent realization, and unsurprisingly most studies, including our own, did not routinely do this a few years ago. Nevertheless, it can help to make sure that the design is suitable to answer the researchers’ hypothesis.

A second recommendation is to use other non-RL approaches to check that an effect that appears in parameters obtained from an RL model is truly present and estimable. One such way is to use a regression approach that can also provide an estimate of the learning rate. Regressions have the advantage of not depending on starting values, local minima or the precise cost function, as is the case for many optimization algorithms. In the case of fitting choice data, a logistic regression model would be appropriate. However, depending on the effects captured by the learning model, it may be necessary to reparametrize the predictors so that they are suitable for a regression analysis. To give an example of what this might entail for the learning rate, we return to Figure 1 which shows that the learning rate captures how much choices were influenced by the outcomes received on the preceding trials. This influence of previous outcomes can be captured by separate regressors that each model the outcome on one of the preceding trials. A learning rate of 1 would mean that only the previous trial’s outcome influences the next choice and is therefore given a non-zero parameter estimate in a regression analysis. In contrast, a smaller learning rate of 0.2 or 0.5 would show non-zero parameter estimates that decrease with increasing distance from the current trial with the largest influence for the outcome on trial t-1, but a still considerable influence of the outcome on trial t-2 and a more diminished influence of the outcome on trial t-3, etc. (Figure 1). We recently used a simple logistic regression (lme2 in R) in addition to a learning model (Lockwood *et al.*, 2019) to show the same effect using two methods, namely, that learning to avoid harm for another person was more model-free than learning to avoid harm for oneself. Similarly, Wittmann *et al*. (2016) showed that RL-derived estimates of performance influenced self and other evaluation; as a control, similar influences were seen without the use of an RL model when doing a regression using the previous history of outcomes. In deterministic associative learning, the % correct can also provide a good approximation of the true underlying learning rate and thus provide a way to validate the model fitting (Lockwood *et al.*, 2018). Simple checks such as the ones outlined in this paragraph are not time-consuming but can help to be confident in the parameter estimates obtained through model fitting.

### Model comparison

To contrast hypotheses, it is sometimes necessary to compare the performance of several models. For example, we might want to test whether the same learning rate is used when learning for oneself *vs* another person (Lockwood *et al.*, 2016, 2018), and thus we want to compare a model with the same learning rate for both agents with a model that uses two separate learning rates. Which of the two models describes a better fit to the data?

**Fig. 5:**
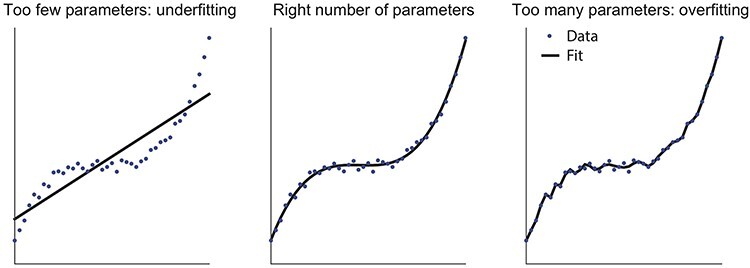
Model complexity when comparing models. Example showing simulated datapoints in blue and three fits (black) ranging from linear (one parameter, left), to cubic (three parameters, middle) to 10th order (10 free parameters, right). This illustrates the concept of underfitting (left), where the model is too simple and does not capture the underlying structure of the data, *vs* overfitting (right) which describes situations in which the model captures not just the structure but also the noise that is specific to the dataset and which will not generalize to new instantiations of the same underlying task structure. The right level of complexity (middle) should capture the structure, but not the noise present in the data. Model comparison can help determine the right level of complexity, but it is a debated topic with multiple options to choose from.

Unfortunately, model comparison is a much-debated topic, with no one-size-fits-all solution. One of the most widely modelled comparison tools is the Bayesian Information Criterion (BIC; Schwarz, 1978). It approximates the Bayes factor (Kass and Raftery, 1995) and is easy to compute as −2 ^*^ log-likelihood + numParams ^*^ ln(nTrials). However, the BIC tends to over-penalize more complex models with additional free parameters and favours simpler models. This helps avoid overfitting—a process whereby too many parameters are used to explain data which can mean not just the structure but the noise is fitted (Figure 5). But it is sometimes overly conservative. On the contrary, sometimes the Akaike Information Criterion is used (AIC; Akaike, 1998). The AIC is computed as −2 ^*^ log-likelihood + 2 ^*^ numParams and has the opposite tendency of preferring overly complex models. For both AIC and BIC, models with small values are preferred over models with larger values. When AIC and BIC agree in their conclusion, it is an easy decision to know which model to prefer. However, sometimes they do not agree in which case it can be a judgement call to know which model to prefer. If there is a specific hypothesis about which parameters are expected to be different, and classical statistics show that those parameters are significantly different, then this can be a reason to favour a model that wins using only one method. Moreover, in the simplest scenario, when the models that are being compared have the same number of parameters, it is sufficient to simply compare them based on their log-likelihood.

Finally, as an alternative to AIC and BIC and other Bayesian methods not described here due to their complexity (Stephan *et al.*, 2009; Penny, 2012; Klein-Flügge *et al.*, 2015, 2016), cross-validation can be used to evaluate the performance of different models. The rationale is quite simple, yet it is a powerful method. The measure of interest is how well a model predicts left-out data and thus how robust and generalizable the prediction from this model is to datapoints that have not influenced the fit. Generally, it is recommended to leave out between a fifth and a tenth of the data in each fold (James *et al.*, 2017). More precisely, the fitting procedure is applied to a subset of the data, e.g. 90% for 10-fold cross-validation. In the cases where there are temporal dependencies between trials, such as in the case of reinforcement learning, all trials can be included during fitting but the negative log-likelihood returned for only 90% of data. This means that the parameter optimization will be performed on 90% of trials. The obtained parameter estimates are then used to predict choices in the left-out 10% of trials, and this procedure is repeated nine more times so that each trial has once been left out and given an out-of-sample prediction. The average log-likelihood of the left-out data given the model parameters can then be used as a measure of model performance and compared across different models (e.g. Zhu *et al*., 2012).

## Summary and future directions

Reinforcement learning models have provided new insights into social cognition and behaviour. Particularly when applied to neuroimaging data, these models can be very powerful and allow the estimation of trial-by-trial changes in the BOLD signal. There are both theoretical and practical considerations when making use of reinforcement learning models including the number of parameters to include in the model, the type of model, the model fitting procedure to use and whether and how to perform a model comparison.

Future studies using a reinforcement learning approach might help to further understand the difference in brain areas tracking social *vs* non-social prediction errors. They might also help to answer one of the fundamental questions in social neuroscience, whether there are brain areas uniquely specialized for social cognition. Moreover, the areas that are considered part of the ‘social brain’ may broaden. For example, there is an emerging role for the subgenual anterior cingulate cortex in social cognition that has mainly come from recent studies showing socially specific prediction error signals in this area (reviewed in Lockwood and Wittmann, 2018), something that would not be possible without a computational approach. It may be that conceptualizing social situations within a reinforcement learning framework can generate new hypotheses about the kinds of social situations humans encounter in everyday life, such as false beliefs about others’ mental states being conceptualized as a prediction error problem (Apps *et al.*, 2013; Koster-Hale and Saxe, 2013). Finally, further studies can start to examine how the different social and non-social signals are integrated to shape our behaviour, perhaps through employing connectivity analyses (Suzuki and O’Doherty, 2020). Overall, we hope this introduction will serve as a useful guide for researchers wishing to use reinforcement learning models in their neuroimaging studies.

## Resources

There are several excellent publicly available resources with example code and tutorials for fitting reinforcement learning models to data including:

A tutorial on fitting RL and Bayesian learning models by Hanneke Den Ouden and Jill O’Reilly:

http://www.hannekedenouden.ruhosting.nl/RLtutorial/Instructions.html.

A practical reinforcement learning course on Coursera:

https://www.coursera.org/learn/practical-rl.

A computational modelling course that covers the methodological considerations explained here in more detail and with the corresponding code, by Miriam Klein-Flügge, Jacqueline Scholl, Laurence Hunt and Nils Kolling:

https://git.fmrib.ox.ac.uk/open-science/computational-models-course.

## Materials and correspondence

Please address correspondence to Patricia L. Lockwood and Miriam C. Klein-Flugge.
